# Serum biomarker discovery related to pathogenesis in acute coronary syndrome by proteomic approach

**DOI:** 10.1042/BSR20210344

**Published:** 2021-06-04

**Authors:** Miji Shin, Sora Mun, Sang Hyun Park, Jiyeong Lee, Hee-Gyoo Kang

**Affiliations:** 1 Department of Senior Healthcare, Graduate School, Eulji University, Seongnam 13135, Korea; 2 Department of Internal Medicine, School of Medicine, Eulji University, Daejeon 34824, Korea; 3 Department of Biomedical Laboratory Science, College of Health Science, Eulji University, Uijeongbu 11759, Korea; 4 Department of Biomedical Laboratory Science, College of Health Sciences, Eulji University, Seongnam 13135, Korea

**Keywords:** acute coronary syndrome, biomarkers, pathogenesis, proteomics

## Abstract

Acute coronary syndrome (ACS) results from inadequate supply of blood flow from the coronary arteries to the heart or ischemia. ACS has an extremely high morbidity and mortality. The levels of biomarkers currently used for detection of ACS also increase in response to myocardial necrosis and other diseases and are not elevated immediately after symptoms appear, thus limiting their diagnostic capacity. Therefore, we aimed to discover new ACS diagnostic biomarkers with high sensitivity and specificity that are specifically related to ACS pathogenesis. Sera from 50 patients with ACS and healthy controls (discovery cohort) each were analyzed using mass spectrometry (MS) to identify differentially expressed proteins, and protein candidates were evaluated as ACS biomarkers in 120 people in each group (validation cohort). α-1-acid glycoprotein 1 (AGP1), complement C5 (C5), leucine-rich α-2-glycoprotein (LRG), and vitronectin (VN) were identified as biomarkers whose levels increase and gelsolin (GSN) as a biomarker whose levels decrease in patients with ACS. We concluded that these biomarkers are associated with the pathogenesis of ACS and can predict the onset of ACS prior to the appearance of necrotic biomarkers.

## Introduction

Acute coronary syndrome (ACS) refers to the clinical symptoms of ischemia or restricted blood supply from the coronary arteries to the heart. Patients with ACS present with acute chest discomfort or pain owing to impaired blood flow to the myocardium [1]. It includes unstable angina (UA), ST segment elevation myocardial infarction (STEMI), and non-STEMI (NSTEMI), according to electrocardiogram (ECG) variations of the ST segment [2,3].

Over 1 million patients with ACS die every year worldwide [1]; thus, rapid and accurate diagnosis is necessary to provide an appropriate treatment that offers good prognosis for the patient [4]. In patients with symptoms, such as acute chest pain or discomfort, correct diagnosis of ACS relies on the confirmation of the ST segment in the ECG and the presence of elevated biomarkers, including the troponin complex (troponin I and troponin T) [2,4,5]. However, the diagnosis is limited by the low sensitivity of the ECG (approximately 50%) [6,7]. The troponin complex is the most sensitive biomarker for acute myocardial infarction (AMI) diagnosis [8]; their levels increasing in blood 4 h after the onset of symptoms. Therefore, the diagnosis of myocardial infarction is limited by the time of patients arrival for treatment [9]. Because of this, the American College of Cardiology/American Heart Association guidelines state to immediately proceed to reperfusion treatment for patients with NSTEMI with persistent symptoms or psychogenic shock, even when the patient has been treated with medication or is suspected to have STEMI by ECG examination or without significant changes in biomarkers [10]. Troponin levels may be elevated not only in coronary artery disease (CAD) such as ACS but also in other diseases that cause myocardial damage [11]; thus, its positive predictive value may be limited. Furthermore, these biomarkers may not be useful to differentiate AMI from UA [12], which amplifies the need for new diagnostic biomarkers with high sensitivity and specificity for diagnosing ACS.

To this end, we focused on the specific pathogenesis underlying plaque rupture-induced ACS, which results from the accumulation of low-density lipoprotein (LDL) carrying cholesterol in the artery. LDL reacts with free radicals and is converted into oxidized LDL (Ox-LDL) inducing inflammation. Then, inflammatory cells, such as macrophages, remove the Ox-LDL, ingest the accumulated cholesterol, and convert into foam cells, which form the arterial plaque [13,14]. In addition, smooth muscle cells (SMCs), endothelial, and phagocytic cells are also induced in the fibrous cap enclosing plaque. Matrix degradation and cell apoptosis of SMC and endothelial cells are promoted by proteases secreted by these cells; thus, arterial inflammation during ACS onset results in matrix degradation, cell apoptosis, and subsequent rupture of the unstable plaque [15,16]. There are studies confirming the prognostic value of inflammatory biomarkers [17], such as C-reactive protein and serum amyloid A [18], but research focusing on ACS pathogenesis at diagnosis has not yet been conducted. Therefore, as inflammation, matrix degradation, and apoptosis, which affect plaque vulnerability, are closely related to the onset of ACS, discovering biomarkers related to these factors could reflect the condition of the patient from the time of plaque formation, well before typical biomarkers, such as troponin, are induced from necrotic myocardium. Early diagnosis of ACS can increase the survival rate as timely appropriate treatment can be pursued.

Although studies on ACS biomarkers and related genes are ongoing [19–21], gene involvement cannot predict the potential effects of alternative splicing and post-translational modifications (PTMs) [22], whereas protein expression may reveal their direct function and relationship to disease [23–25]. Hence, the importance of proteomics, the identification, quantity, structure elucidation, and interaction of the expressed proteome *in vivo*, which can be used to facilitate identification of protein biomarkers for screening, diagnosis, staging, prognosis, and treatment [26,27]. In proteomics research, mass spectrometry (MS) is widely used to analyze complex biological samples and discover biomarkers of various diseases [24], such as rheumatoid arthritis and ischemic stroke [28,29].

**Figure 1:**
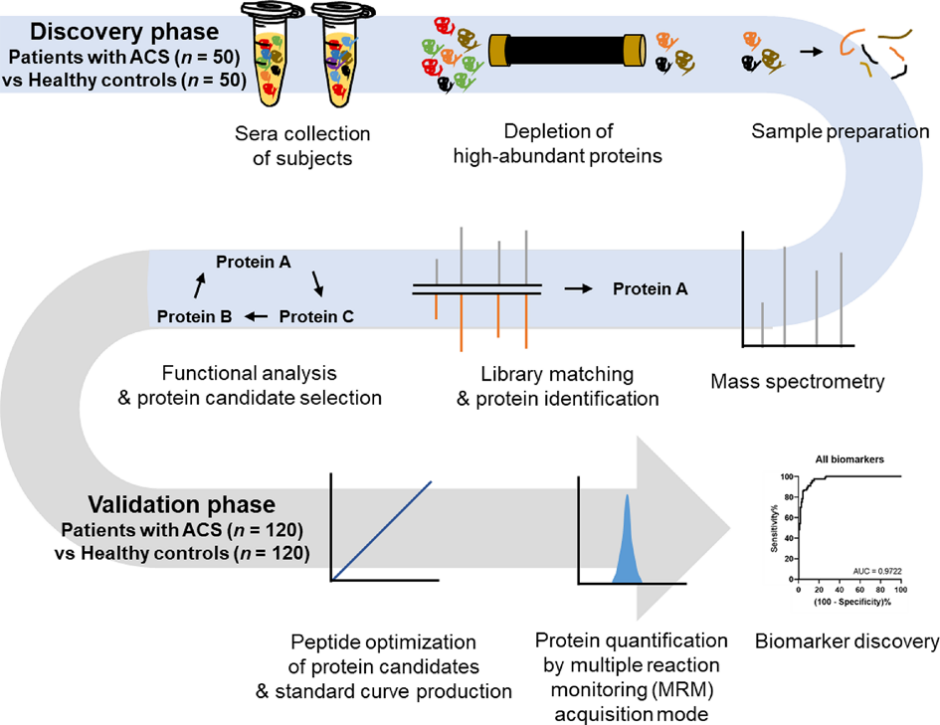
Flowchart of experiment set Sera of 50 patients with ACS and 50 healthy controls consisted of pooled and individual serum samples, respectively, and the samples were prepared for MS. The pooled serum samples were analyzed in information-dependent acquisition (IDA) mode to generate a library of proteins. In addition, protein identification was performed by matching the data of individual serum samples analyzed with sequential window acquisition of all theoretical fragment-ion spectra (SWATH) mode to the library. Peptides from each protein were quantified using MRM acquisition mode for validation of biomarkers identified from candidate proteins.

In the present study, we analyzed the sera of patients with ACS and healthy controls using MS. Specifically, we have assessed the significant differential expression of identified proteins and performed functional analysis to identify and validate early diagnostic biomarkers of inflammation, matrix degradation, and apoptosis, related to the onset of ACS by plaque rupture. Additionally, these early diagnostic biomarkers were validated by performing quantification through multiple reaction monitoring (MRM) acquisition mode (Figure 1).

## Materials and methods

### Human sera collection

Human sera were collected from Eulji University Hospital Institutional Review Board from 2017 to 2020 (approval EMC 2016-03-019); all the enrolled participants provided written informed consent. All patients were admitted to the hospital for symptoms of ACS, such as chest pain and heart attack, diagnosed through ECG, and blood test. We collected sera from a total of 50 patients with ACS and 50 healthy controls to assess the presence of novel potential protein biomarkers for ACS early diagnosis, and the sera of 120 patients with ACS and 120 healthy controls to validate the protein candidates (Table 1). Patients with ACS were confirmed diagnostically, and the healthy controls had no CAD. Fresh whole blood samples were collected in vacutainers without anticoagulant at 25°C, and centrifuged at 4000×***g*** for 5 min. The sera were obtained from supernatant and stored at −80°C until evaluated.

**Table 1:** Demographic information of the enrolled subjects

Parameter	Discovery cohort		Validation cohort	
	Patients with ACS (*n*=50)	Healthy controls (*n*=50)	Patients with ACS (*n*=120)	Healthy controls (*n*=120)
Gender (female/male)	10/40	10/40	43/77	51/69
Age^*^ (years)	62.4 ± 13.1	56.8 ± 4.7	63.4 ± 13.5	53.3 ± 6.8
BMI^*^ (kg/m^2^)	24.4 ± 3.4	23.8 ± 2.4	24.8 ± 3.4	24.3 ± 2.5
Blood pressure^*^ (systolic/diastolic)	129.1 ± 21.3/78.5 ± 13.4	126.7 ± 13.7/77.8 ± 10.4	131.6 ± 23.6/79.2 ± 14.2	125.4 ± 13.8/76.6 ± 10.5
Smoking (no/yes)	27/23	18/32	57/63	70/50
Diabetes mellitus (no/yes)	33/17	50/0	72/48	117/3
Hyperlipidemia (no/yes)	25/25	48/2	48/72	117/3
Hypertension (no/yes)	22/28	50/0	53/67	120/0
Familial CAD (no/yes)	48/2	46/4	117/3	110/10
Previous CABG (no/yes)	50/0	N/A	119/1	N/A
Previous PCI (no/yes)	42/8	N/A	96/24	N/A
LVEF^*^ (%)	53.8 ± 10.9	N/A	54.6 ± 11.0	N/A
CK-MB^*^ (ng/ml)	32.6 ± 70.2	N/A	38.5 ± 66.1	N/A
CRP^*^ (mg/dl)	0.3 ± 0.3	N/A	0.8 ± 2.1	N/A
Glucose^*^ (mg/dl)	161.1 ± 76.2	91.4 ± 9.8	160.5 ± 75.8	94.0 ± 21.6
LDL^*^ (mg/dl)	101.2 ± 35.7	128 ± 30.1	101.4 ± 37.4	126.6 ± 31.8
Total cholesterol^*^ (mg/dl)	169.1 ± 36.0	208.9 ± 31.3	168.8 ± 39.3	208.4 ± 34.0
Triglyceride^*^ (mg/dl)	144.1 ± 97.9	120.2 ± 50.0	154.0 ± 94.1	142.0 ± 84.3
Troponin T^*^ (ng/ml)	600.5 ± 2398.8	N/A	1.3 ± 2.1	N/A

Abbreviations: CABG, coronary artery bypass graft; CK-MB, creatinine kinase-myocardial band; LVEF, left ventricular ejection fraction; PCI, percutaneous coronary intervention.

^*^Mean ± standard deviation.

### Sample preparation

Individual serum samples were diluted 1:3 with multiple affinity removal system buffer A (1.2% sodium chloride and 0.02% sodium azide in high-performance liquid chromatography (HPLC) grade water (Sigma–Aldrich, St. Louis, MO, U.S.A.) and centrifuged at 15000×***g*** for 1 min using a membrane filter (Spin-X Centrifuge Tube Filter 0.22-μm; Corning, Salt Lake, UT, U.S.A.). We eliminated high-abundance proteins (albumin, antitrypsin, haptoglobin, IgA, IgG, and transferrin) with a multiple affinity removal system LC-column (human 6, 4.6 × 50 mm; Agilent Technologies, Santa Clara, CA, U.S.A.) at 4°C for 18 min. Low-abundance protein samples were centrifuged at 4°C and 12000×***g*** for 25 min using Nanosep® centrifugal device (molecular weight cutoff (MWCO) 3000; Pall Corporation, Port Washington, NY, U.S.A.), then dried in ScanSpeed 40 coupled with Teflon (LaboGene, Denmark).

Concentrated protein samples were dissolved with lysis buffer (8 M urea and 0.1 M Tris-HCl, pH 8.5), and the concentration was determined by the Bradford method (Pierce, Rockford, IL, U.S.A.). The individual and pooled serum samples were diluted to 100 μg and 1 mg, respectively. Further, 5 M Tris (2-carboxyethyl) phosphine (Pierce, Rockford, IL, U.S.A.) was added and the samples were shaken at 37°C for 30 min, followed by addition of 50 mM ammonium bicarbonate (Sigma–Aldrich, St. Louis, MO, U.S.A.) to adjust the pH to 8.3. Iodoacetamide (0.5 mM) (Sigma–Aldrich, St. Louis, MO, U.S.A.) was applied and the samples were agitated at 25°C for 1 h without light. Then, they were iterated to add 50 mM ammonium bicarbonate to adjust pH. Finally, Trypsin Gold (Mass spectrometry grade; Promega, Madison, WI, U.S.A.) was added to digest proteins overnight at 37°C. Samples were adjusted to pH under 3.0 with 10% formic acid (Sigma–Aldrich, St. Louis, MO, U.S.A.). Cleanup was conducted using C18 cartridges (Waters, Milford, MA, U.S.A.), and the samples were dried in ScanSpeed 40 coupled with Teflon.

The above protein extraction and processing process were carried out for pooled serum samples as well as for individual serum samples. Additionally, OFFGEL electrophoresis was conducted with the pooled serum samples using OFFGEL Fractionator (Agilent Technologies). The samples were separated by pI using OFFGEL strip (Immobiline® DryStrip, pH 3-10; GE Healthcare, Madison, WI, U.S.A.).

### MS

The samples were analyzed using the Nano-LC system Ekspert nLC415 (Eksigent Technologies, Dublin, CA, U.S.A.) coupled to the AB SCIEX 5600 triple TOF mass spectrometer (AB SCIEX, Concord, Canada). The LC solvents used were mobile phase solution A (0.1% formic acid in HPLC-grade water) and mobile phase B (0.1% formic acid in HPLC-grade acetonitrile). Next, 2 μl of samples were injected into the NanoLC trap column (0.5 mm × 350 µm; 3 µm; Eksigent Technologies) and eluted from the analytic column (150 mm × 75 µm; 3 µm; Eksigent Technologies) to the nanospray tip (PicoTip Emitter Silica Tip by New Objective, Woburn, MA, U.S.A.) to ionize. Total gradient time was 120 min, during which the mobile phase solution B was 5–40% for the first 105 min, followed by 40–90% for 0.5 min, 90% for 6 min, 90 to 5% for 0.5 min, and 5% for the last 8 min, at a constant flow rate of 300 nl/min. Further, 50 fM β-galactose was analyzed once for every three samples as auto calibration. The parameters were set to 12 ion source gas, 25 curtain gas (CUR), 2200 V ion spray voltage floating, and 150°C interface heater temperature. Data were analyzed in positive ion mode.

### Information-dependent acquisition mode to generate a library from pooled serum samples

The pooled serum samples were analyzed using the information-dependent acquisition (IDA) mode. The MS scan range setting was 250–2000 mass-to-charge ratio (m/z) for precursor ions. The top ten of precursor ions have +2 to +5 charges and over ten counts per cycle were selected. The MS/MS scan range setting was 100–2000 m/z for product ions. Using the ProteinPilot v.5.0 search engine (AB SCIEX), a protein dataset was obtained from the human UniProt Swiss-Prot database (released April 2017). The parameters were set to cys-alkylation (iodoacetamide), digestion (trypsin with allowing for two missed cleavages), instrument (TripleTOF 5600), and species (*Homo sapiens*). A library of proteins was generated using pooled serum samples data.

### Sequential window acquisition of all theoretical fragment-ion spectra mode to generate spectra data from individual serum samples

The individual serum samples were analyzed by sequential window acquisition of all theoretical fragment-ion spectra (SWATH) mode. The isolation width was 20 Da (window overlapping; 1 Da), and a total of 53 overlapped windows were covered with the MS scan range of 250–1250 m/z and the MS/MS scan range of 100–2500 m/z. Collision energy was automatically adjusted for each window.

### Statistical and functional analyses for protein candidate selection

In PeakView v.2.2 (AB SCIEX), peptides were relatively quantified using the library from the ProteinPilot group file by matching their acquisition mass and retention time. The false discovery rate (FDR) of peptides was lower than 1%; modifications and shared peptides were excluded. Normalized data were obtained by using the total area sums normalization of the MarkerView v.1.3.1 software (AB SCIEX). Student’s *t* test and principal component analysis (PCA) were used to compare differently expressed proteins between the patients with ACS and healthy controls.

We used String Database v.11.0 (Search Tool for Retrieval of Interacting Genes/Proteins; https://string-db.org/) to rank the pathways based upon −log (FDR).

Before validating ACS early diagnostic biomarkers by MRM, the peptide of protein candidates corresponding to the following conditions were selected in PeakView: peptides without (1) N-terminal methionine owing to oxidation; (2) miscleaved trypsinization; (3) modification; and peptides with (4) sequence length of 15 amino acids or less. Two subjects of each gender from each group were selected randomly among the total subjects, and proteins satisfying all conditions in all eight subjects were identified as protein candidates.

### Quantification of protein candidates, validation through MRM acquisition mode, and statistical analysis

A standard peptide for each protein candidate was selected by checking the intensity and FDR, and were synthesized with a purity of ≥90% (Peptron, Daejeon, South Korea). In addition, to determine Q1/Q3 ion pairs for target peptide quantification, optimization was performed through Skyline (http://proteome.gs.washington.edu/software/skyline) and the AB SCIEX QTRAP® (Triple quadrupole linear ion trap) 5500 system (AB SCIEX). Entrance potential and collision cell exit potential were set to positive, 10 and 11 V, respectively. A standard curve for each peptide was constructed using the selected analysis conditions for optimization. Sera from patients with ACS and healthy controls were analyzed with standard curves, and the protein candidates in the sera were quantified using AB SCIEX QTRAP® 5500 equipped with ACQUITY ultra performance liquid chromatography (UPLC) BEH C18 column (130 Å, 1.7 µm, 2.1 mm × 150 mm, Waters) and ACQUITY UPLC BEH C18 VanGuard pre-column (130 Å, 1.7 µm, 2.1 mm × 5 mm, Waters). The analysis was performed with a total gradient time of 30 min, which is 5–90% mobile phase B; a flow rate of 0.25 µl/min was maintained using 5 µl of sample. During the gradient time, the mobile phase solution B was 10–15% for the first 1 min, followed by 15–40% for 19 min, 40–90% for 1 min, 90% for 4 min, 90 to 10% for 0.5 min and 5% for the last 4.5 min. LC solvents were mobile phase solution A (0.1% formic acid in HPLC-grade water) and mobile phase B (0.1% formic acid in HPLC-grade acetonitrile). The source parameters for the acquisition method were set to 40 and 60 ion for gas source 1 and 2, respectively; 30 CUR; 5500 V ion spray voltage; and 400°C temperature in positive ion mode. Normalization was performed using an internal standard.

The quantitative data of each protein was analyzed by scatter plot and receiver operating characteristic (ROC) curve, generated using the GraphPad Prism v.8.4.2 software (San Diego, CA, U.S.A.). We determined data distribution with the Kolmogorov–Smirnov test and statistical significance with Student’s *t* test or Mann–Whitney U test. In addition, the area under the curve (AUC) was confirmed via multiple logistic regression by combining the date on confirmed biomarkers.

## Results

### Protein identification between pooled and individual serum samples

**Figure 2:**
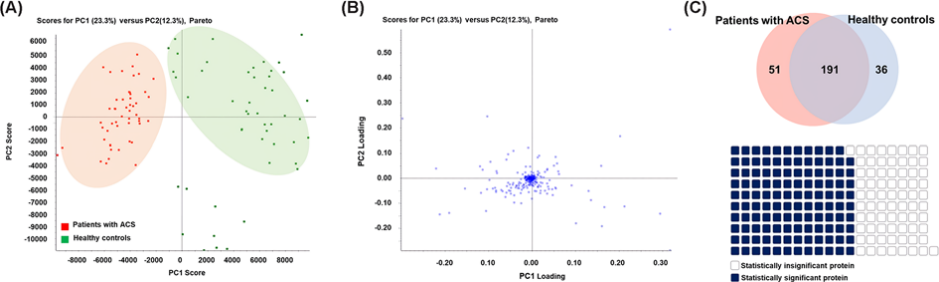
Proteomic analysis of proteins from patients with ACS and healthy controls (**A**) The PCA score plot showed clustering of patients with ACS and healthy control groups. Each dot indicates patients with ACS (red) and healthy controls (green). (**B**) The loading plot showed proteins from patients with ACS and healthy controls. The proteins helped distinguish each group of patients with ACS and healthy controls in the PCA score plot. (**C**) A total of 242 and 227 proteins were identified in patients with ACS and healthy controls, respectively, and 191 proteins were overlapped between the groups. Therefore, these 191 proteins were common to all subjects. Using *t* test, we identified 119 statistically significant proteins out of 191 differentially expressed proteins between patients with ACS and healthy controls (*P*-value < 0.05, fold change ≥1.2 or ≤1/1.2).

Pooled serum sample data obtained through the IDA mode and individual serum sample data obtained through the SWATH mode were matched. To confirm that protein identification progressed properly with accuracy, a PCA score plot was constructed to confirm the clustering of each group (Figure 2A). The loading plot showed that the proteins properly calculated as a variable for each group (Figure 2B). Additionally, a Venn diagram was generated to select common proteins among all the subjects. A total of 242 proteins in the patients with ACS, 227 proteins in the healthy controls, and 191 proteins common to both groups were identified. Of the 191 overlapping proteins, 119 proteins showed significant differential expression (*P*-value <0.05, fold change (patients with ACS/healthy controls) ≥1.2 or ≤1/1.2) by Student’s *t* test (Figure 2C).

### Screening of significant proteins

To screen for statistically significant proteins, proteins with *P*-value of less than 0.05, fold change ≥1.2 or ≤1/1.2, were selected through Student’s *t* test, yielding 119 proteins. Additionally, 33 proteins with peak intensity ≥ 1000 and FDR < 1% were selected in PeakView (Table 2).

**Table 2:** List of significant proteins selected through PeakView

No.	Uniprot accession number	Protein name	Expression	*P*-value	Fold change
1	P02763	α-1-acid glycoprotein 1	Up	5.28E^−09^	1.66
2	P01011	α-1-antichymotrypsin	Up	2.53E^−08^	1.26
3	P43652	Afamin	Up	1.30E^−04^	1.25
4	P02656	Apolipoprotein C-III	Up	1.94E^−02^	1.22
5	Q13790	Apolipoprotein F	Up	4.03E^−03^	1.59
6	P10909	Clusterin	Up	1.20E^−11^	1.41
7	P0C0L4	Complement C4-A	Up	4.30E^−04^	1.22
8	P0C0L5	Complement C4-B	Up	6.20E^−05^	1.38
9	P01031	Complement C5	Up	4.72E^−13^	1.24
10	P10643	Complement component C7	Up	3.50E^−04^	1.21
11	P07360	Complement component C8 γ chain	Up	3.31E^−03^	1.25
12	P02748	Complement component C9	Up	1.30E^−08^	1.36
13	P08603	Complement factor H	Up	3.12E^−11^	1.43
14	P02790	Hemopexin	Up	8.08E^−09^	1.24
15	P04196	Histidine-rich glycoprotein	Up	2.50E^−03^	1.58
16	Q06033	Inter-α-trypsin inhibitor heavy chain H3	Up	2.12E^−10^	1.56
17	Q14624	Inter-α-trypsin inhibitor heavy chain H4	Up	5.32E^−11^	1.22
18	P02750	Leucine-rich α-2-glycoprotein	Up	8.50E^−09^	1.64
19	P02775	Platelet basic protein	Up	1.90E^−04^	1.45
20	P02760	Protein AMBP	Up	2.21E^−06^	1.51
21	Q9UK55	Protein Z-dependent protease inhibitor	Up	7.67E^−06^	1.28
22	P05543	Thyroxine-binding globulin	Up	1.20E^−04^	1.22
23	P04004	Vitronectin	Up	2.22E^−06^	1.35
24	P25311	Zinc-α-2-glycoprotein	Up	5.70E^−07^	1.27
25	P02765	α-2-HS-glycoprotein	Down	1.02E^−15^	0.62
26	P06727	Apolipoprotein A-IV	Down	9.50E^−03^	0.83
27	O14791	Apolipoprotein L1	Down	5.30E^−10^	0.64
28	P08185	Corticosteroid-binding globulin	Down	4.55E^−10^	0.63
29	P02751	Fibronectin	Down	9.90E^−12^	0.33
30	P06396	Gelsolin	Down	2.11E^−08^	0.81
31	P01871	Immunoglobulin heavy constant mu	Down	3.34E^−14^	0.04
32	P00734	Prothrombin	Down	3.11E^−05^	0.77
33	P05452	Tetranectin	Down	7.89E^−11^	0.74

### Functional analysis of differentially expressed proteins

To confirm the function of the 33 selected proteins, biological process (gene ontology (GO)) and molecular function (GO) analyses of the proteins was conducted through the STRING database. In the biological process (GO) analysis, the 33 differentially expressed proteins were associated with the processes of inflammation and matrix degradation such as regulation of proteolysis (FDR = 1.27E^−20^%), regulation of peptidase activity (FDR = 1.68E^−14^%), negative regulation of endopeptidase activity (FDR = 1.70E^−14^%), and negative regulation of proteolysis (FDR = 3.45E^−14^%). In addition, the regulation of acute inflammatory response was in the top ten biological processes (FDR = 8.60E^−14^%). Similarly, in the molecular function (GO) analysis, peptidase regulator activity (FDR = 3.07E^−15^%), endopeptidase inhibitor activity (FDR = 6.54E^−15^%), enzyme inhibitor activity (FDR = 1.18E^−12^%), enzyme regulator activity (FDR = 4.26E^−11^%), serine-type endopeptidase inhibitor activity (FDR = 1.19E^−10^%), lipid binding (FDR = 1.50E^−04^%), and cytokine receptor binding (FDR = 4.88E^−02^%) were also found to be related to differentially expressed proteins (Table 3).

**Table 3:** List and related functions of protein candidates

No.	Uniprot accession number	Protein name	Expression	*P*-value	Fold change	Biological process (GO) rank^*^										Molecular function (GO) rank^†^									
						1	2	3	4	5	6	7	8	9	10	1	2	3	4	5	6	7	8	9	10
1	P02763	α-1-acid glycoprotein 1	Up	5.28E^−09^	1.66								√												
2	P01031	Complement C5	Up	4.72E^−13^	1.24	√	√	√	√	√	√	√		√	√	√	√	√	√	√			√	√	√
3	P02750	Leucine-rich α-2-glycoprotein	Up	8.50E^−09^	1.64																		√	√	√
4	P04004	Vitronectin	Up	2.22E^−06^	1.35	√	√	√	√	√		√		√	√						√		√	√	
5	O14791	Apolipoprotein L1	Down	5.30E^−10^	0.64																	√			
6	P06396	Gelsolin	Down	2.11E^−08^	0.81	√		√			√													√	
7	P05452	Tetranectin	Down	7.89E^−11^	0.74	√		√					√								√				

^*^The ranks of the biological process (GO): regulation of proteolysis (rank 1), regulation of humoral immune response (rank 2), regulation of protein processing (rank 3), regulation of complement activation (rank 4), regulation of protein activation cascade (rank 5), regulation of peptidase activity (rank 6), negative regulation of endopeptidase activity (rank 7), platelet degranulation (rank 8), negative regulation of proteolysis (rank 9) and regulation of acute inflammatory response (rank 10). **^†^**The ranks of the molecular function (GO): peptidase regulator activity (rank 1), endopeptidase inhibitor activity (rank 2), enzyme inhibitor activity (rank 3), enzyme regulator activity (rank 4), molecular function regulator (rank 5), heparin binding (rank 6), lipid binding (rank 7), signaling receptor binding (rank 8), protein binding (rank 9) and cytokine receptor binding (rank 10).

**Figure 3:**
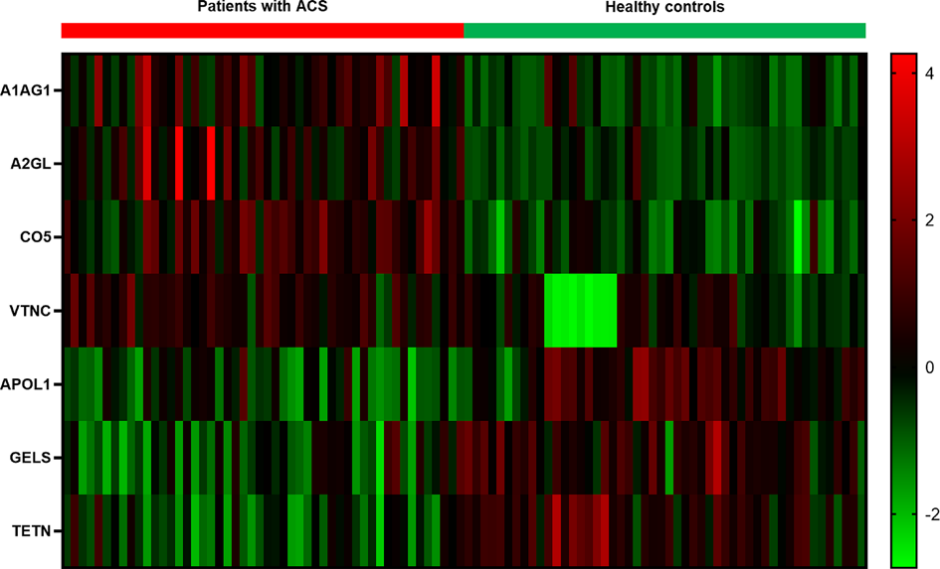
Heat map of the protein candidates between patients with ACS and healthy controls The heat map showed the difference in expression of protein candidates between patients with ACS and healthy controls using Z-score. AGP1, complement C5, leucine-rich α-2-glycoprotein, and vitronectin were up-regulated proteins in patients with ACS. Apolipoprotein L1, gelsolin, and tetranectin were down-regulated proteins in patients with ACS; A1AG1, α-1-acid glycoprotein 1; A2GL, leucine-rich α-2-glycoprotein; CO5, complement C5; VTNC, vitronectin; APOL1, apolipoprotein L1; GELS, gelsolin; TETN, tetranectin.

Through conditional filtering in MRM, we selected seven proteins as potential ACS early diagnostic biomarkers. The candidate proteins were validated with the functional analysis (Table 3) and are shown in the heat map using Z-score (Figure 3). α-1-acid glycoprotein 1 (AGP1), complement C5 (C5), leucine-rich α-2-glycoprotein (LRG), and vitronectin (VN) were up-regulated in patients with ACS, whereas apolipoprotein L1, gelsolin (GSN), and tetranectin were down-regulated.

### ACS early diagnostic biomarker identification

**Figure 4:**
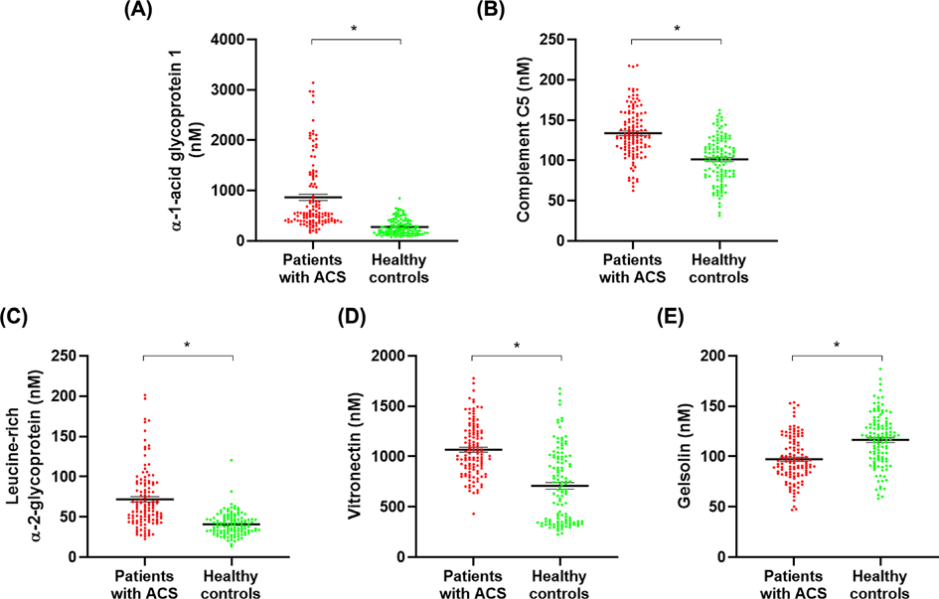
Scatter plots of ACS early diagnostic biomarkers Five biomarkers were selected as proteins showing the same expression in the discovery and validation set. The scatter plots indicate expression of biomarkers in patients with ACS and healthy controls. (**A**) AGP1, (**B**) complement C5, (**C**) leucine-rich α -2-glycoprotein, and (**D**) vitronectin were up-regulated proteins in patients with ACS. In contrast, (**E**) gelsolin was the down-regulated protein in patients with ACS. The difference in the expression of all biomarkers was statistically significant. Data are presented as mean ± SD. *P<0.001.

**Figure 5:**
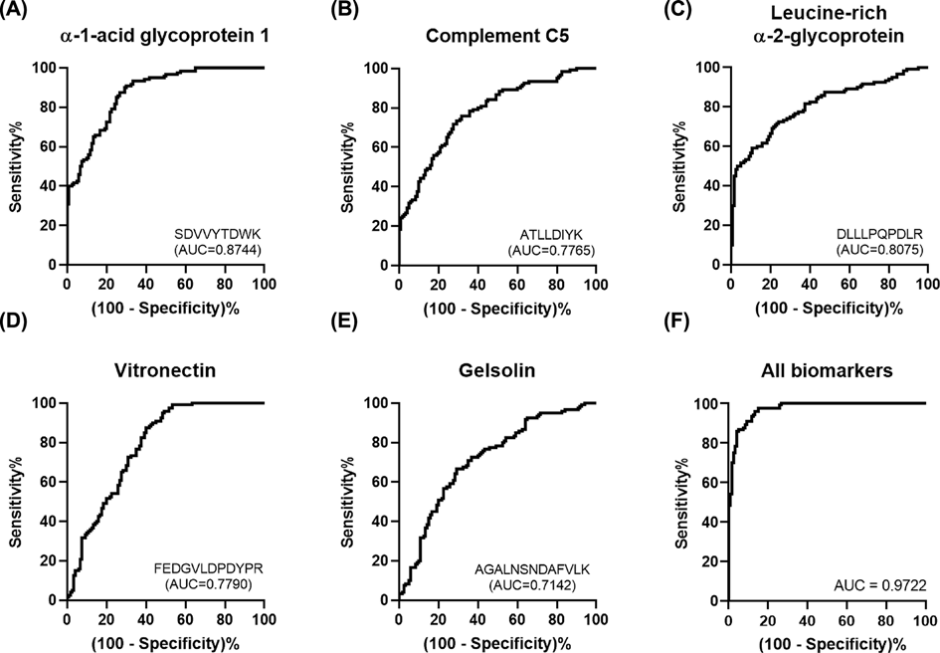
ROC curves of ACS early diagnostic biomarkers The ROC curves showed the AUC of ACS early diagnostic biomarkers. (**A**) The AUC of AGP1 was 0.8744. (**B**) The AUC of complement C5 was 0.7765. (**C**) The AUC of leucine-rich α-2-glycoprotein was 0.8075. (**D**) The AUC of vitronectin was 0.7790. (**E**) The AUC of gelsolin was 0.7142. All ACS early diagnostic biomarkers had AUC higher than 0.7. (**F**) The AUC of all combined biomarkers was 0.9722, which displayed the highest result.

The candidate proteins were analyzed and quantified in MRM acquisition mode for validation. The parameters were set to generate three divalent or trivalent charged product ions per precursor ion, and collision energy, declustering potential, and retention time suitable for each standard peptide were selected as analysis parameters through the optimization process (Table 4). Only one parameter in each standard peptide was selected for quantification (Table 4). Five of the seven candidate proteins showed significant results and were confirmed to have the same expression between groups in scatter plots (Figure 4). All AUCs also showed a significant value at 0.7 or higher (Figure 5). Consequently, these five proteins were selected as ACS early diagnostic biomarkers, and it was confirmed that they were related to the pathogenesis of ACS through other studies (Table 5).

**Table 4:** List of MRM acquisition mode parameters for biomarker discovery

No.	Uniprot accession no.	Protein name	Peptide sequence	Target ion	Q1 (m/z)	Q3 (m/z)	RT (min)	DP (V)	CE (V)
1	P02763	α-1-acid glycoprotein 1	SDVVYTDWK	2b3	556.767	302.135	4.41	71.7	28.9
				2y5	556.767	712.330	4.41	71.7	28.9
				**2y6**	**556.767**	**811.398**	**4.41**	**71.7**	**28.9**
2	P01031	Complement C5	ATLLDIYK	**2y2**	**468.774**	**310.176**	**7.25**	**65.3**	**25.7**
				2y4	468.774	538.287	7.25	65.3	25.7
				2y6	468.774	764.455	7.25	65.3	25.7
3	P02750	Leucine-rich α-2-glycoprotein	DLLLPQPDLR	**2b2**	**590.340**	**229.118**	**8.99**	**74.2**	**30.1**
				2b3	590.340	342.202	9.00	74.2	30.1
				2y6	590.340	725.394	9.00	74.2	30.1
4	P04004	Vitronectin	FEDGVLDPDYPR	2b2	711.830	277.118	5.90	83.0	34.5
				**2y5**	**711.830**	**647.315**	**5.90**	**83.0**	**34.5**
				2y7	711.830	875.426	5.90	83.0	34.5
5	O14791	Apolipoprotein L1	VTEPISAESGEQVER	2y6	815.900	717.353	3.07	90.6	38.2
				**2y8**	**815.900**	**933.427**	**3.08**	**90.6**	**38.2**
				2y10	815.900	1091.496	3.08	90.6	38.2
6	P06396	Gelsolin	AGALNSNDAFVLK	2b3	660.351	200.103	6.13	79.3	32.6
				**2y2**	**660.351**	**260.197**	**6.12**	**79.3**	**32.6**
				2y9	660.351	1007.516	6.13	79.3	32.6
7	P05452	Tetranectin	LDTLAQEVALLK	2b3	657.387	330.166	10.10	79.0	32.5
				2y7	657.387	800.488	10.10	79.0	32.5
				**2y8**	**657.387**	**871.525**	**10.10**	**79.0**	**32.5**

The **bold-faced** target ion was used in final quantification. Abbreviations: CE, collision energy; DP, declustering potential; RT, retention time.

**Table 5:** The ACS early diagnostic biomarkers related to pathogenesis of ACS

No.	Protein name	Pathogenesis		
		Inflammation	Matrix degradation	Apoptosis
1	α-1-acid glycoprotein 1	√	√	
2	Complement C5	√	√	
3	Leucine-rich α-2-glycoprotein	√	√	√
4	Vitronectin	√		√
5	Gelsolin	√		√

## Discussion

ACS is a disease with high morbidity and mortality but unfortunately its current diagnostic biomarkers such as troponin cannot be detected at the early stages of ACS, and their levels may be elevated in other diseases that present with myocardial damage. Thus, the use of these biomarkers for ACS diagnosis has its limitations [9,11]. To address this deficiency, we aimed to discover novel ACS diagnostic biomarkers, focusing on the pathogenesis associated with plaque rupture, which induces ACS. Considering that plaque rupture can be induced by inflammation, matrix degradation, and apoptosis. The identification of diagnostic biomarkers associated with these factors before the onset of ACS, could be a valuable tool for ACS early diagnosis, resulting in its promptly treatment. To discover biomarkers, we validated the proteins that showed significantly differential expression between the patients with ACS and the healthy controls, and then proceeded with the functional analysis. We confirmed that these differentially expressed proteins were related to ACS pathogenesis, and thus we selected them as biomarker candidates and validated this result through quantification.

AGP1, also called orosomucoid, is a highly glycosylated protein, and with molecular weight of 44 kDa, of which 45% is carbohydrate [30,31]. This protein is mainly secreted by hepatocytes, and its expression is regulated by pro-inflammatory cytokines such as tumor necrosis factor (TNF) and actinomycin D (ActD) [30,32]. During an acute phase response, it increases two- to seven-times in serum [31,32], inducing an anti-inflammatory response, especially by promoting antineutrophil activity [33]. By suppressing secretion from neutrophils, a plaque-induced inflammatory cell, AGP1 may inhibit the release of enzymes that degrade matrix components such as matrix metallopeptidases (MMPs) [34]. In addition, since it is an essential protein for suppressing platelet aggregation activated by collagen or thrombin stimulation, it is possible that AGP1 would also suppress plaque rupture [35]. Furthermore, AGP1 levels in patients with ACS were found to be higher than in healthy controls [36]. As a result, it can be inferred that pro-inflammatory cytokines are induced in the production of intravascular plaque, and AGP1 is increased to suppress the subsequent inflammation by targeting neutrophils. Consistent with other studies, AGP1 levels were increased in ACS patients, and applying AGP1 to the diagnostic criteria can identify associated inflammation and matrix degradation before plaque rupture (Table 5). It was also confirmed that the AUC was 0.8744, which was the highest among the biomarkers (Figure 5A).

C5, whose molecular weight is 188 kDa, belongs to the complement system that induces inflammatory response and cell death. This molecule is activated by proteolytic enzymes released from leukocytes, thrombin, kallikrein, apoptotic cells, and modified LDL in plaque [37,38]. When activated, it induces cell death by dividing into the pro-inflammatory molecules C5a and C5b [39], generating an inflammatory response and the formation of a membrane attack complex (MAC, C5b-9) on the cell membrane [40]. Our results revealed that C5 levels were increased in patients with ACS, and other studies have shown that C5 further induced inflammation in existing inflammatory conditions [41]. Additionally, HDL suppresses pro-inflammatory cleavage fragments, such as C3a and C5a, and MAC, inducing anti-inflammatory responses and suppressing cell death, respectively [42]. However, since apolipoprotein A-IV, a constituent of HDL, decreased, it can be inferred that apoA-IV was degraded by enzymes such as MMP-14 secreted from inflammatory cells (Table 2) [43]. Therefore, HDL presumably also has reduced activity and cannot suppress C5 properly. In addition, in atherosclerotic lesions, C5a is continuously produced from C5 during complement activation to form the pro-inflammatory polypeptide anaphylatoxin [44]. C5a binds to and activates its receptor on endothelial cells, SMCs, lymphocytes, monocytes, granulocytes and macrophages [44,45]. Macrophages and SMC express plasminogen activator inhibitor-1 (PAI-1), a major inhibitor of fibrin degradation, to inhibit elimination of thrombi produced by plaque rupture [38]. Macrophages also express MMP-1 and MMP-9 to degrade the matrix [46]. Furthermore, it has been reported that C5a is not induced in stable plaques and only increases in unstable plaques [46], which suggests that C5a may have a key role in the pathogenesis inducing plaque rupture. In addition, similar to the results of our study, it has been reported that plaque generation from patients with AMI and atherosclerosis increased C5 levels [47–49]. An increment in C5 level may be related to inflammation and matrix degradation, which induce the onset of ACS (Table 5), and as shown in Table 3, C5 was found in the top ten functions and processes such as complement activation, inflammation response, and proteolysis activity. Thus, C5 may also be useful for early diagnosis of ACS.

LRG is a protein with a molecular weight of approximately [50] associated with cell adhesion, granulocytic differentiation, and cell migration [51–53]. LRG is produced and secreted by endothelial cells, neutrophils, macrophages, and hepatocytes, and is induced by cytokines such as interleukin-6 (IL-6) during inflammatory reaction [54–56], other inflammatory diseases also increase LRG levels [57]. Furthermore, LRG level has a positive correlation with IL-6 and also may be associated with the differentiation of inflammatory T lymphocytes [58]. This T lymphocytes produce and secrete granzyme, a serine protease that degrades extracellular matrix (ECM) components such as collagen, fibronectin, and proteoglycan [15]. In addition, T cells express granzyme and perforin to induce apoptosis of target cells such as SMC and endothelial cells [59]. From this, LRG levels may be increased by the inflammatory reaction resulting from the production of plaque, and this increased LRG level induces matrix degradation and cell apoptosis in ACS. In fact, another study has found that patients with AMI tend to have increased LRG levels compared to healthy controls [60]. Additionally, Table 3 shows that LRG is related to a cytokine–receptor binding function associated with inflammation induction, regulation of proteolysis, peptidase regulator activity, and enzyme regulator activity as is involved in matrix degradation. Thus, LRG is associated with inflammation, matrix degradation, and apoptosis in the pathogenesis of ACS (Table 5). Moreover, because the AUC was 0.8075 (Figure 5C), if LRG serum levels increase, ACS may develop; thus, early diagnosis and treatment are possible.

VN presents in plasma and ECM, with a molecular weight of approximately 75 kDa [61], binds to collagen, complement, and heparin [62]. Furthermore, VN, which is expressed in the vascular endothelium or SMC in response to the production of plaque, is involved in a variety of biological processes such as angiogenesis, regulation of cell–ECM interphase, tissue remodeling, and modification of endothelial tissue [61–64]. The cytokine-induced increase in VN level during the inflammatory reaction is stabilized by binding to PAI-1. The PAI-1/VN complex suppresses the mechanism by which plasminogen is converted to plasmin; therefore, the complex inhibits the degradation of thrombi formed by plaques [65,66]. Further, VN participates in cell adhesion by binding to integrin α_V_ß_3_ within the ECM, but PAI-1 blocks the activity of integrin α_V_ß_3_; hence, cell proliferation is suppressed, and apoptosis is induced [65,67]. It was determined that VN levels increased compared to healthy controls in studies on AMI and CAD [61,63]. The functions shown in Table 3 are also found to be associated with the pathogenesis of ACS. In addition, since VN is associated with inflammation and apoptosis, it could possibly be used for early diagnose of ACS.

GSN, widely expressed in a variety of tissues including the heart, brain, and immune cells, is approximately 84 kDa, and is an actin-regulating protein that plays an important role in cell motility [68–70]. This protein regulates macrophage function and plays a significant role in the process of inflammation; specifically, it suppresses inflammation by inhibiting IL-6 and TNF-α secretion from macrophages [68,69]. In addition, GSN blocks inflammatory responses and also secretion of cytochrome C from mitochondria to the cytosol to suppress apoptosis [71], and regulates physiological processes such as signal transduction in various diseases [69]. Since the GSN level of patients with ACS decreased, inflammation caused by macrophage activity and apoptosis caused by cytochrome C, may contribute to disease in patients with ACS. The results of our study as well as other studies showed the same tendencies in patients with ACS, with lower GSN levels than in healthy controls [72]. Thus, decreased GSN levels may result in a high risk of ACS, which could possibly be used in ACS early diagnosis.

To discover diagnostic biomarkers related to these pathogeneses, we validated proteins differentially expressed in patients with ACS and healthy controls, and selected those related to inflammation, matrix degradation, and apoptosis. We found that the five biomarkers were associated with plaque rupture, and with LRG in particular, and have a multifaceted relationship with three pathogenesis of plaque rupture. Sensitivity and specificity were increased when the biomarkers identified in our study were used in combination while examining their correlation with ACS, with an AUC of 0.9722, which was improvement in comparison with their individual values (Figure 5F). The combined evaluation of these biomarkers may enable ACS early diagnosis, before its onset.

The identified biomarkers were associated with the pathogenesis of plaque rupture and thus could enable early diagnosis, which is an improvement on the current traditional necrosis-induced biomarkers. Early identification will allow the physicians to begin treatment before ACS onset, preventing its development. The results showed that the biomarkers identified in this study can be clinically utilized; however, they require clinical validation for its use in ACS diagnosis, along with ECG examination and currently used biomarkers. Moreover, further study of these biomarkers might lead to their use not only in early diagnosis but also in the prevention of ACS-induced plaque formation and rupture. In addition, a follow-up study will be conducted to confirm the association between the pathogeneses of plaque rupture and the biomarkers discovered here, and to identify biomarkers that could be used to diagnose specifics ACS, such as STEMI, USTEMI, and UA.

## Perspectives

- ACS results from inadequate supply of blood flow from the coronary arteries to the heart, or ischemia, and presents ahigh worldwide morbidity and mortality. The biomarkers currently available for ACS evaluation increase in response to myocardial necrosis and other diseases and thus are limited in its diagnostic capacity. Moreover, they are not elevated immediately after the symptoms appear. Therefore, we aimed to discover new ACS diagnostic biomarkers with high sensitivity and specificity that are particularly related to plaque rupture pathogeneses.
- Proteins differentially expressed in patients with ACS and healthy controls were identified and further validated as diagnostic biomarkers. AGP1, C5, LRG, and VN levels were increased in patients with ACS, and GSN levels were decreased.
- These biomarkers are related to ACS pathogeneses and can predict the onset of ACS prior to the appearance of the necrotic biomarkers currently used.

## Data Availability

The data that support the findings of the present study are available from the corresponding authors upon reasonable request.

## Abbreviations

ACS: acute coronary syndrome
ActD: actinomycin D
AGP1: α-1-acid glycoprotein 1
AMI: acute myocardial infarction
AUC: area under the curve
C5: complement C5
CAD: coronary artery disease
CUR: curtain gas
ECG: electrocardiogram
ECM: extracellular matrix
FDR: false discovery rate
GO: gene ontology
GSN: gelsolin
HPLC: high-performance liquid chromatography
IDA: information-dependent acquisition
IL-6: interleukin-6
LDL: low-density lipoprotein
LRG: leucine-rich α-2-glycoprotein
MAC: membrane attack complex
MMP: matrix metallopeptidase
MRM: multiple reaction monitoring
MS: mass spectrometry
NSTEMI: non-ST segment elevation myocardial infarction
Ox-LDL: oxidized low-density lipoprotein
PAI-1: plasminogen activator inhibitor-1
PCA: principal component analysis
QTRAP: triple quadrupole linear ion trap
ROC: receiver operating characteristic
SMC: smooth muscle cell
STEMI: ST segment elevation myocardial infarction
SWATH: sequential window acquisition of all theoretical fragment-ion spectra
TNF: tumor necrosis factor
UA: unstable angina
UPLC: ultra performance liquid chromatography
VN: vitronectin
